# Validation of the extended e-health literacy scale: structural validity, construct validity and measurement invariance

**DOI:** 10.1186/s12889-024-19431-8

**Published:** 2024-07-25

**Authors:** Gregor Petrič, Sara Atanasova

**Affiliations:** Center for Methodology and Informatics, Faculty of Social Sciences, University of Ljubljana, Kardeljeva ploščad 5, Ljubljana, 1000 Slovenia

**Keywords:** E-health literacy, Measure, Scale, Validity, Measurement invariance

## Abstract

**Background:**

Given the rapid proliferation and use of online health resources, many of which may be of dubious quality, there is an increasing need to develop electronic health literacy (e-health literacy) skills among the population of internet users. E-health literacy encompasses the skills and abilities needed to access, understand, validate, evaluate, interpret, and apply online health-related information. Measuring e-health literacy has become crucial for developing targeted interventions, assessing their impact, and producing high-quality research findings that can inform health policy and clinical practice, which can lead to improved health outcomes and potentially reducing health inequalities. The scales need to be valid and reliable so that decisions are based on high-quality data. In this regard, the issue of the measurement invariance of scales across different demographic groups has been neglected. This is critical, as assessments should be valid across different sociodemographic groups to avoid bias when comparing them. The aim of this study was to validate the Extended e-health literacy scale (eHEALS-E) on general population and investigate its structural validity and internal consistency, construct validity in terms of convergent and discriminant validity, and examine its measurement invariance across gender, age, education and social status.

**Methods:**

The data were collected as a part of a national health literacy survey conducted by the Slovenian National Institute of Public Health. For this survey the initial eHEALS-E scale was revised in order to address its limitations and applicability to general population. Based on a nationally representative sample, the final sample for the analysis comprised 1,944 individuals who at least occasionally used one of the various internet services to obtain health-related information. Multiple group confirmatory factor analysis was used to examine the measurement invariance of the scale.

**Results:**

With some adjustments, the measurement model of the revised 6-dimensional eHEALS-E scale demonstrated a good fit to the data (χ^2^ = 2508, df = 282, RMSEA = 0.064, SRMR = 0.070, CFI = 0.90). The scale had good internal consistency (alpha = 0.89). Although evidence of the scale’s convergent and discriminant validity was partially provided, the analysis revealed robust measurement invariance across sociodemographic groups.

**Conclusions:**

With a minor limitation, the scale ensures an unbiased e-health literacy assessment across different social groups, which is crucial for interventions that aim to reduce health-related social inequalities. This ensures that the interventions derived from the assessment of reality are equally valid and effective for everyone, regardless of their sociodemographic background.

## Background

Given the abundance of diverse health-related information available online and the information overload in the healthcare field, exacerbated by the COVID-19 pandemic, there is a growing need for the skills and abilities required to access, understand, validate, evaluate, interpret, and apply online health-related information. This information can often aid decision-making in managing health problems, [1] but this is not without risk: information is not always credible, reliable, or accurate and can lead people to make problematic decisions and misdiagnoses with significant public health consequences [2, 3]. It is therefore not surprising that, in the last decade, research has increasingly focused on assessing electronic health literacy (e-health literacy) in different populations and contexts. [4–6] E-health literacy refers to the ability to find, understand, and evaluate health-related information obtained using electronic resources and to use such information in managing and solving health problems [7]. Similar to health literacy, [8, 9] e-health literacy is considered to play a critical role in achieving better health outcomes, health-promoting behaviors, improved communication skills in doctor‒patient relationships, and patient empowerment [10–12].

One of the ongoing challenges in this research area is the valid and reliable scale of e-health literacy [13–15]. Such tools are not only important for accurately assessing the distribution of e-health literacy in different populations. They are also critical for healthcare providers developing tailored communication strategies with patients, addressing any deficiencies, ultimately leading to improved health outcomes and potentially reducing inequalities in access to health information and services [16]. In addition, such tools are important for developing targeted interventions, measuring their impact, and producing high-quality research findings that can inform health policy and practice [11]. As the World Health Organization (WHO) points out in its Global Strategy on Digital Health 2020–2025, it is essential to measure e-health literacy to track its progress over time; this calls for the development of valid and reliable e-health literacy scales that can be used in different cultures and settings. Among these scales, Norman and Skinner’s eHealth Literacy Scale (eHEALS), [7] in which was coined the term e-health literacy, is one of the most commonly used measures [6, 16–18]. However, this scale with 8 to 10 items developed to be used in clinical settings has often been criticized for being unidimensional and unable to assess the different dimensions of the concept (accessing, understanding, appraising, and applying the online information that is relevant to health), which has consequently led to the weaknesses in quality of measuring and low validity of the scale [19–21]. In addition, the eHEALS scale has been often criticized for being outdated in the context of today’s landscape of various internet-based information retrieval applications and services. Accordingly, it has become inadequate for measuring competencies and skills needed in the context of technological advancements, more complex and interactive digital information environment (14, 20–21). Due to these shortcomings of the eHEALS, several new scales of e-health literacy have been proposed: the DHLI, [22] e-HLS, [23] eHLA, [24] eHLQ, [25] TeHLI, [26], etc. This also includes the Extended e-health literacy scale (eHEALS-E), which was developed and validated to overcome the limitations of the eHEALS [27]. The scale that includes additional items (20 items in total) improves and extends the eHEALS and measures the dimensions of e-health literacy more comprehensively, is less subject to social desirability bias, and addresses the skills to critically evaluate the biases and limitations of the digital technologies and social media used for health-related purposes [27]. It is based on the expanded definition of e-health literacy [27] and has in comparison to eHEALS demonstrated empirical multidimensional structural validity with six meaningful dimensions: [14, 27] Awareness of various online sources, Validating online information, Recognizing the quality and meaning of online information, Perceived efficiency, Being smart on the Net, and Understanding online information.

However, while the eHEALS-E scale was validated in a population of online health community users, [27] when first introduced, its validity in the general population remains unknown. Although eHEALS-E scale has demonstrated good structural validity, it needs to be validated on general population. Further evidence of different types of validity is also needed, along with addressing the somewhat problematic internal consistency of the seminally introduced scale. Moreover, as previous studies have shown that there are several differences in e-health literacy levels between different population groups, especially in terms of gender, age, education, and social status [28–30] it is important to ascertain that the scale measures the same phenomenon for all these groups. For example, men were found to have a greater level of e-health literacy than women, [31, 32] although this result is inconsistent. [28] Furthermore, older adults generally report lower levels of e-health literacy; [11] higher levels of education correlate positively with higher levels of e-health literacy [33, 34]. Moreover, lower social status has been associated with lower levels of health literacy [35] as well as e-health literacy [36]. This suggests a lack of e-health literacy among the already underprivileged population in the healthcare field. However, these studies have rarely investigated whether the measurement properties of the e-health literacy scale are the same for different sociodemographic groups; moreover, observed differences can appear due to measurement bias. It is thus important to examine the measurement invariance of the e-health literacy scale to determine whether a scale behaves similarly across different subpopulations [37].

Thus, the aim of this study was to validate the eHEALS-E scale on general population by investigating its structural validity, internal consistency, construct validity, and measurement invariance across sociodemographic groups (gender, age, education, and social status). To achieve this aim we have first revised the original eHEALS-E scale to enhance its applicability to the general population and its relevance in contemporary e-health contexts. This study thus aims to address the following research question: What is the structural validity, internal consistency, construct validity in terms of convergent and discriminant validity, and measurement invariance across sociodemographic groups (gender, age, education, and social status) of the eHEALS-E scale?

## Methods

### Data collection and procedure

The data were collected within WHO Action Network on measuring Population and organizational Health Literacy (M-POHL), where national implementation and data collection was organized by the Slovenian National Institute of Public Health (SNIPH) as part of the National Health Literacy Survey in Slovenia [38]. The target population of the research consisted of adults (18 years and older) living in Slovenia. The sample was provided by the Statistical Office of the Republic of Slovenia using two-stage stratified random sampling based on the Central Population Register. The data were collected between March and August 2020. The individuals in the sample were invited to participate in a web survey based on postal invitation. Those who did not participate in the web survey were offered the option to complete the survey via a computer-assisted personal interview method or on paper and return it by post.

Of the 5,585 individuals contacted, 3,360 adequately completed the questionnaire, resulting in a 60% response rate. The analysis for this study was limited to a subsample of individuals who at least occasionally used one of the various internet services to obtain health-related information. More specifically, respondents received six questions that pertain to the frequency of using search engines, Facebook pages, online forums in Slovenia and abroad, specialized health-related sites in Slovenia and abroad and other online health-related services for obtaining health-related information (see Table 1 for distribution of responses). Those who responded with answers 1–4 on a scale of 1–5 (1-every day, 2-several times per week, 3-several times per month, 4-less than once per month, 5-never) on at least one item, received the battery of items of the eHEALS-E scale. There were 2,238 (66.6%) individuals who met these criteria. After listwise deletion of missing units in the variables in the analysis, the final sample size for the analysis was *n* = 1,944.

**Table 1:** Frequency of using various internet services to obtain health-related information (*n* = 1944)

	Every day	Several times per week	Several times per month	Less than once per month	Never
Search engines (i.e. Google, Bing, Yahoo)	27,2%	14,9%	25,4%	30,1%	2,4%
Facebook pages related to health	8,2%	8,2%	13,5%	17,5%	52,6%
Online forums in Slovenia (i.e. Med.Over.Net, Ringaraja.net)	1,2%	5,5%	16,3%	33,4%	43,6%
Online forums abroad	1,5%	2,2%	6,3%	21,4%	68,6%
Specialized national websites for health-related issues (i.e. Nijz.si, Vizita.si, Zdravje.si)	1,3%	6,4%	22,3%	40,8%	29,2%
Specialized foreign websites for health-related issues	0,6%	2,6%	8,2%	21,9%	66,5%
Other health-related online services	0,8	2,5	4,9	10,9	80,9

The sample (see Table 2) consists of 55.1% of females, those who are between 36 and 45 years old represent the largest age group (22.8%), while the most respondents completed higher education (43.2%). Most of the respondents were employed (65.4%). The analysis of social status reveals that 18.8% of respondents perceived themselves as having low social status, 70.0% as medium and 11.2% as high social status.

**Table 2:** Sample characteristics (*n* = 1944)^1^

Variable		*n*	%
Gender	Male	872	44.9
	Female	1072	55.1
Age	18–25	233	12.0
	26–35	380	19.5
	36–45	444	22.8
	46–55	377	19.4
	56–65	323	16.6
	66–75	146	7.5
	75+	41	2.1
Education	Primary school or less (ISCED Levels 0–2)	319	16.4
	Secondary school (ISCED Level 3)	786	40.4
	Higher education (ISCED Levels 5–8)	839	43.2
Social status^2^	Low	342	18.8
	Medium	1347	70.0
	High	216	11.2
Employment status	Employed, self-employed, farmer	1271	65.4
	Homemaker, unable to work, unemployed	179	9.2
	Retired	342	17.6
	Student	152	7.8

^1^ Sample size for Social status is *n* = 1925 due to missing values on this variable

^2^ Social status was assessed using a 10-point scale ranging from 1 (lowest position in society) to 10 (highest position in society). An index of 1 to 4 was defined as low social status; values from 5 to 7 were categorized as medium social status; a score greater than 8 was defined as high social status [35]

### Revision of the initial extended e-health literacy scale (eHEALS-E)

The eHEALS-E scale was first introduced in a study of online health community users [27]. In addition to the suggestion that validating a scale on a different population always demands a revision of the items, [39] the original scale was also revised for two additional reasons: (a) The internal consistency of the Validating information and Being smart on the Net dimensions was rather problematic; [27] (b) The protocol for constructing questionnaire by SNIPH demanded several steps that allowed us to revise and actualize the scale. The revision of the scale thus comprised the following steps: (1) Evaluation of the original scale by an expert panel and proposal of revised/new items; (2) Computation of content validity index for all items; (3) Review of all items by experts from SNIPH; (4) Cognitive interviews on a selection of items; (5) Final battery of items to be included in the questionnaire.

To explain these steps in more detail, the first step comprised of a panel of three experts in public health, social psychology, and social science methodology that assessed the original scale for relevance and clarity. Among the total number of 20 items in the original scale, 11 of them remained the same, while 9 of them were changed to improve clarity and relevance. Such change can be exemplified with a modification of item on the Recognizing quality and meaning dimension, where the item from the original scale “I can tell high-quality from low-quality health resources on the Internet.” was modified to “I am able to distinguish low-quality health information from high-quality health information online.”. The change emphasizes the active process of discerning information quality, rather than the outcome, and addresses the variability of quality within the same sources, thus more accurately capturing the complexities of navigating online health resources and the critical evaluation in this process.

In addition, new 1–3 items per each dimension were developed in order to enlarge the pool of items for further expert review, resulting in 32 items altogether. For example, the dimension of Validating information was enhanced with the item “When I find information related to my health online, I check its accuracy with other online sources.”, as this aspect of cross-validating information was not grasped by the original scale. The dimension of Being smart on the Net included two new items (“Online systems are so highly developed that they automatically differentiate between low- and high-quality health information.” and “A large number of followers (of a person or an organization) on social media is a proof that information posted online is professionally reliable.”). Disagreement with these statements indicates the respondent’s critical awareness of internet algorithms and that popularity of content do not necessarily equate to information quality [40].

A pool of all 32 items was evaluated again by the same experts as in the previous step in order to calculate the content validity index (I-CVI) [41]. The three experts provided responses to the question “How essential is this item to the construct being measured?” on a scale from 1 (not essential) to 4 (very essential). I-CVI was then calculated as the number of evaluators giving a rating of 3 or 4, divided by the total number of evaluators. The two items that had CVI-I lower than 1.0 (full agreement) were further discussed and refined in order to reach the full agreement.

Another round of reviews was conducted by experts from SNIPH, who gave comments on the clarity and grammar of the items, and on this basis the items were further improved. Of all 32 items, 11 were assessed in cognitive interviews, organized by SNIPH, which examined the understanding and difficulty of items and appropriateness of the scale’s responses. Interviews were conducted with 7 members of the target population with different backgrounds in terms of gender, education, age, region and health status. Based on the cognitive interview results, slight adjustments were made to the wording of several items.

### Measures

#### Extended e-health literacy scale (eHEALS-E)

In this research a revised version of eHEALS-E was used, based on the outcome of the above described revision process. In the questionnaire, the items were introduced with the question: “In this section, we focus on your experience using the internet to search for health-related information. Please rate on a scale from 1 (does not apply at all) to 5 (applies completely), to what extent the following statements apply to you.” The scale was originally deployed in Slovenian, while here, we present the English translation, which was conducted by SNIPH. For a list of all the items, see Table 3.

**Table 3:** Confirmatory factor analysis (CFA) and internal consistency measures of the extended e-health literacy scale (eHEALS-E)

Scale items	Awareness of Sources	Validating Information	Recognizing Quality	Perceived Efficiency	Smart on the Net	Understanding Information
I know which sources of health information are available online.	0.66					
There are medical studies published online, but I do not know how to access them. (R)^1^	0.40					
I know how to access websites or applications and enter my symptoms to get information about my health issues.	0.54					
I know how to use the internet to get answers to my health concerns.	0.83					
I know where to find useful sources of information on health online.	0.85					
When I find information related to my health online, I check its accuracy with other online sources.		0.73				
It is important for me to check health-related information that I find online with other sources (for example doctors, books, friends, relatives).		0.58				
If I have doubts about the reliability of information about health online, I ask somebody for explanation.		0.51				
When finding information about health online, I take sufficient time to truly understand it.		0.58				
I am able to distinguish low-quality health information from high-quality health information online.			0.70			
I have no difficulties understanding the substance of the information online.			0.76			
I have sufficient knowledge to assess the quality of online sources.			0.75			
I can identify useful tips for addressing my health issues from information online.			0.78			
I feel confident about using the internet to improve my health.				0.75		
The internet is very useful for helping me to take decisions about my health.				0.73		
It is very important for me to have access to health-related sources online.				0.69		
I know how to use the information I find on the internet to improve my health.				0.77		
I think we can trust most of the health information found online. (R)					0.65	
I am satisfied with the first health source found on the internet that provides answers to my questions. (R)					0.71	
When searching online, I prefer to read short and simple health explanations rather than comprehensive professional explanations. (R)					0.54	
Online systems are so highly developed that they automatically differentiate between low- and high-quality health information. (R)					0.73	
A large number of followers (of a person or an organization) on social media is a proof that information posted online is professionally reliable. (R)					0.71	
I often do not understand the terminology used by some online health sources. (R)						0.67
I sometimes have difficulties understanding key information online that is relevant to my health. (R)						0.75
I’m unable to recognize high-quality information relevant for my health because of the vast amount of information online. (R)						0.76
I fully understand health-related information I find online.						0.64
**Cronbach’s alpha**	0.77	0.69	0.85	0.82	0.80	0.79
**Composite reliability (omega)**	0.77	0.70	0.81	0.82	0.80	0.80

^1^ (R) denotes a reversed item, suggesting that agreement with the item indicates absence of e-health literacy, while disagreement indicates presence of e-health literacy

#### Short health literacy scale

The short HLS-EU-Q12 scale that can be used as a substitute for the longer 47-item multidimensional health literacy scale [38] has demonstrated good measurement properties. Cronbach’s alpha is 0.82, while the confirmatory factor analysis (CFA) of the 12 items demonstrates a good fit to the data (SRMR = 0.04, RMSEA = 0.02, CFI, TLI; GFI = 1.00). An example item is “How difficult is it for you to act on advice from your doctor or pharmacist?” The scale ranged from 1 (very easy) to 4 (very difficult).

#### Navigational health literacy scale

The navigational health literacy scale [42] pertains to the difficulties experienced in the tasks related to accessing, understanding, appraising, and applying information to navigate the healthcare system. The 4-point rating scale ranges from 1 (very difficult) to 4 (very easy). Cronbach’s alpha (0.90) demonstrated excellent internal consistency, and the CFA shows a good fit to the data (SRMR = 0.05, RMSEA = 0.05, CFI, TFI, GFI = 0.99). An example item from the 12-item scale is “How difficult is it for you to judge to what extent your health insurance covers a particular health service?”.

### Analytic procedure

Structural validity refers to the extent to which the items of a scale represent the underlying dimensionality of the construct [43] and was investigated with CFA, exploratory factor analysis (EFA) and internal consistency statistics. For assessing the CFA model fit, a root mean square error of approximation (RMSEA) less than 0.08, a standardized root mean square residual (SRMR) less than 0.08, and a comparative fit index (CFI) greater than 0.90 were considered acceptable [44]. The values of Cronbach’s alpha (α) and the composite reliability (CR; also known as McDonald’s omega) were used to assess the internal consistency, with values > 0.70 defined as acceptable [37, 44].

Construct validity refers to the extent to which the items of a scale accurately measure the intended characteristics [45]. Empirically, construct validity is determined by convergent and discriminant validity [45]. Convergent validity, which is concerned with the empirical similarity between the scales of theoretically related constructs, is tested for the eHEALS-E in relation to health literacy and navigational health literacy, which are conceptually related to the concept of e-health literacy [38]. While health literacy is the basis of both concepts, navigational health literacy refers to the ability to effectively navigate health systems and services to ensure that one has access to the appropriate health services for one’s health problems and needs [38, 46]. Convergent validity was assessed by calculating Pearson’s correlation coefficients between eHEALS-E and scales of health literacy and navigational health literacy, as theoretically related measures [47]. Following Cohen’s criteria of effect size, it is suggested that one can speak of the convergent validity if the correlation with the theoretically related variable shows at least a medium effect size (*r* > .3) [48].

Discriminant validity, on the other hand, focuses on whether the scores obtained from an assessment of a particular construct remain unique and are not influenced by other constructs [49]. It was assessed using the square root of the average variance extracted (AVE), which was expected to be higher than the correlations of the scale with other scales in the model [37].

A measurement model with good structural validity was used to examine the measurement invariance across four sociodemographic variables: gender, age, education, and social status. The measurement invariance was tested with the multiple group CFA, which is an approach that assesses whether a given construct is measured in the same way across different groups. The testing involves a series of increasingly restrictive models to evaluate whether the parameters of the factor model are equivalent across groups [44]. First, the fit of the configurational models that assumes an equal structure of 6 dimensions was assessed. In the next step, constraints were introduced to test the metric invariance (assuming equal factor loadings) and assess the decrease in fit. The final step was to test a model with equal intercepts (scalar invariance). In this test, if the fit of the model with more constraints is not worse than the fit of the model with fewer constraints, the measurement invariance is supported [44]. To assess the change in model fit, the χ^2^ difference test was performed. However, because this test is sensitive to sample size and violations of normality assumptions, [50] we also observed differences in other metrics. ΔCFI < 0.1 and ΔSRMR < 0.03 indicate measurement invariance [51].

All the analyses were conducted in R using the packages *psych* and *lavaan*.

## Results

### Structural validity and internal consistency of the eHEALS-E

The initial CFA for all 32 items and the assumed six dimensions showed a rather poor fit (χ^2^ = 6320.7, df = 449, RMSEA = 0.083, SRMR = 0.106, CFI = 0.763). Thus, we conducted an EFA with all 32 items, which suggested a six-factor solution as theoretically proposed. However, we found that some items on the dimensions Validating information (e.g., “If I find useful information on health online, I am not interested in who the author is.”), Recognizing quality and meaning (e.g., “I can find a lot of health information online, but I cannot identify the information that can help me make health decisions.”), and Perceived efficiency (e.g., “I do not usually find personally useful information about health online.”) had factor loadings lower than 0.4 and/or communalities lower than 0.2 and were therefore removed from the analysis. One item of Understanding information (“When reading information about health online, I take sufficient time to truly understand it.”), loaded on the Validating information dimension. Therefore, we ran the CFA again, assigning one item to a different dimension and removing 6 items. This time, the measurement model (see Table 3 for the list of all items) demonstrated an acceptable fit to the data (χ^2^ = 2508, df = 282, RMSEA = 0.064, SRMR = 0.070, CFI = 0.90). The analysis of Cronbach’s alphas and McDonald’s omegas (see Table 3) shows that all the dimensions except one have values above 0.7, demonstrating good internal consistency. Only the dimension Validating information had a somewhat borderline value of alpha = 0.69 and omega = 0.70, which is still acceptable but indicates the need for further revision of this dimension.

Although the scale has clear and distinct dimensions, it can also be used as a single scale of overall e-health literacy, as the Cronbach’s alpha for all items is 0.89. Overall e-health literacy is thus a composite measure, grasping the content of all six dimensions, computed as an arithmetic mean of all 26 items.

### Convergent validity

Pearson’s correlation coefficients between the eHEALS-E scale dimensions and two criterion variables (see Table 4) show that health literacy correlated most strongly with overall e-health literacy (*r* = .418, *p* < .001) and the dimensions of Recognizing quality and meaning (*r* = .382, *p* < .001) and Understanding information (*r* = .361, *p* < .001). A similar pattern of correlations can be observed for navigational health literacy, but the correlations are somewhat weaker. The correlations with Being smart on the Net stand out, as this dimension is not correlated with health literacy (*r* = .014, NS) and navigational health literacy (*r*=-.069, *p* = .003).

**Table 4:** Correlations between eHEALS-E dimensions and overall e-health literacy and two criterion variables

Dimensions of eHEALS-E and Overall e-health literacy	Criterion variables		
	Health literacy	Navigational health literacy	
Awareness of Sources	0.293^**^	0.291^**^	
Validating Information	0.227^**^	0.136^**^	
Recognizing Quality	0.382^**^	0.394^**^	
Perceived Efficiency	0.294^**^	0.199^**^	
Smart on the Net	0.014	− 0.069^*^	
Understanding Information	0.361^**^	0.362^**^	
Overall e-health literacy	0.418^**^	0.349^**^	

*Note* ** *p* < .001, * 0.05 < *p* < .001

### Discriminant validity

The square root of the AVE is in most cases larger than the correlations with the other dimensions, except in the case of the correlation between the dimensions Awareness of sources and Recognizing quality and meaning (see Table 5). In this case, the square root of the AVE of the Awareness of sources is 0.64, while the correlation with the Recognizing quality and meaning dimension is 0.66. The correlation between these two dimensions is quite high, indicating that they are not well discriminated.

**Table 5:** Square root of AVE on diagonals, Pearson’s correlations between eHEALS-E scale dimensions off the diagonal

Dimensions of eHEALS-E scale	Awareness of Sources	Validating Information	Recognizing Quality	Perceived Efficiency	Smart on the Net	Understanding Information
Awareness of Sources	0.64					
Validating Information	0.40	0.62				
Recognizing Quality	0.66	0.44	0.75			
Perceived Efficiency	0.50	0.41	0.53	0.74		
Smart on the Net	− 0.16	0.15	− 0.02	− 0.24	0.69	
Understanding Information	0.22	0.14	0.48	0.28	0.28	0.71

### Measurement invariance

The results provide strong evidence of configural invariance for the eHEALS-E scale across gender groups, as the measurement model with an equivalent 6-dimensional latent structure across genders fits the data well (χ^2^ = 2906, df = 564, CFI = 0.89, RMSEA = 0.065, SRMR = 0.073). The results also provide evidence of metric invariance, since the model with introduced constraint of equal factor loadings did not statistically significantly fit worse than the original multigroup model. This is because the difference in χ^2^ was not significant (*p* = .34), and the changes in the CFI and SRMR were small enough to support metric invariance (ΔCFI < 0.1 and SRMR < 0.03). The model with equal intercepts had a significantly worse fit in terms of χ^2^, but the differences in CFI (< 0.1) and SRMR (< 0.01) support scalar invariance. Thus, the evidence shows that the factor structure, factor weights, and intercepts are the same when we measure the eHEALS-E in men or women.

The same procedure was used for education, where three groups were compared (primary school or less, secondary school, higher education), for age, where also three groups were compared (18–35, 36–55, 56+), and similar for social status, where groups with low, middle and high social status were compared). In all three cases, the configuration equivalence can be confirmed (see Table 6), as the configuration models demonstrate a good fit to the data. In addition, both metric and scalar invariance for gender, age, education, and social status can be confirmed, as the stricter models consistently demonstrate the decrease in goodness-of-fit measures within the suggested thresholds (ΔCFI < 0.1 and SRMR < 0.03).

**Table 6:** Summary of measurement invariance comparisons

	Type of invariance	χ^2^	df	RMSEA	SRMR	CFI	*P*(Δχ2)
No groups model		2508	282	0.064	0.070	0.90	/
Gender	Configurational	2906	564	0.065	0.073	0.89	/
Gender	Metric	2928	584	0.064	0.073	0.89	0.34
Gender	Scalar	2991	604	0.064	0.074	0.89	< 0.001
Education	Configurational	3067	846	0.064	0.071	0.89	/
Education	Metric	3149	886	0.063	0.074	0.89	0.01
Education	Scalar	3288	926	0.063	0.075	0.89	< 0.001
Age	Configurational	3231	846	0.066	0.074	0.89	/
Age	Metric	3294	886	0.065	0.076	0.89	0.01
Age	Scalar	3446	926	0.065	0.076	0.88	< 0.001
Social status	Configurational	3159	846	0.065	0.072	0.89	/
Social status	Metric	3235	886	0.064	0.075	0.89	0.001
Social status	Scalar	3354	926	0.064	0.075	0.89	< 0.001

## Discussion

This is the first study to empirically test the eHEALS-E scale on a representative sample of the national population and to comprehensively analyze its structural validity, internal consistency, convergent and discriminant validity, and measurement invariance across a set of sociodemographic groups. The results show that the revised eHEALS-E scale is valid and reliable enough to measure e-health literacy, a crucial skill in today’s information-saturated online environment. We were able to confirm the distinct 6-dimensional structure of the eHEALS-E scale, as previously shown in a specific population of online community users, [27] with the Being smart on the Net dimension standing out as a very distinct dimension. This dimension has no common space with other dimensions and criterion variables, which could be because it is most strongly associated with digital literacy, encompassing abilities and skills needed to navigate dynamic and complex digital information environment [52, 53]. However, this dimension may also be the least susceptible to social desirability bias, as it consists of items that elicit self-reporting of practices and beliefs, whereas self-reporting of skills is more likely susceptible to Dunning-Krueger effect [54]. However, this is only speculation that would need to be empirically investigated by controlling for the scales that measure social desirability bias, such as the Balanced Inventory of Desirable Responding Scale [55]. In any case, it is be argued that the Being smart on the Net dimension is particularly important in the context of the rapidly evolving internet technologies based on artificial intelligence algorithms that require critical awareness, distance, and understanding [56] for an individual to receive accurate and credible online health information.

The other five dimensions of the eHEALS-E scale also provide distinct perspectives on e-health literacy; there is some overlap between the dimensions of Awareness of sources and Recognizing quality and meaning of online information. It seems that the latter may be conditioned by the awareness of the sources that provide quality health information. In other words, individuals who are e-health literate are already aware of quality online health sources, and the process of quality recognition is already preceded by the selection of such sources. Nevertheless, we believe that, in addition to the overall e-health literacy scale, differentiated insight into six dimensions is valuable. For example, high performance in one dimension does not necessarily indicate high performance in another. The study of e-health literacy of online health community users discovered that there is one cluster of users (“core relational users”), which has satisfactory e-health literacy in terms of awareness of sources, perceived efficiency and recognizing quality and meaning, but problematic in terms of validating, understanding information and being smart on the Net [27]. People might be aware and efficient in finding online health information, but having blind trust and not validating it can lead to problematic decisions. Dimensional understanding of e-health literacy and identification of specific gaps between them is thus needed for a more nuanced approach in addressing challenges presented by the online information landscape.

While the initially introduced eHEALS-E scale had low and marginal reliability of the Validating information and Being smart on the Net dimensions, [27] the revised eHEALS-E, validated here, shows improved reliability for these dimensions. There is still a minor issue with the measurement quality of the Validation information dimension, as we found marginal internal consistency and relatively low correlations with the criterion variables. Epistemologically, the items for this dimension are somewhat specific, as they are designed as activities (checking accuracy with other sources, asking for explanations) and not as attitudes or beliefs, as in other dimensions. As they are activities, we could argue that they represent a formative concept for which the calculation of internal consistency is usually not meaningful [57].

The study results provide insight into the measurement invariance of the eHEALS-E across four sociodemographic variables, which is an important contribution to confirming the content validity of the scale [58]. When comparing disadvantaged groups with other groups, it is important that the scale measures the same construct [59]. Given the diversity of Internet users using online health-related information in terms of varied demographic backgrounds, [60, 61] the measurement invariance of the e-health literacy scale is even more crucial. The results suggest that the eHEALS-E can be used for valid comparisons of different sociodemographic groups. Using a scale that guarantees unbiased comparisons is important for gaining the trust of users and stakeholders [20].

### Practical implications

Measuring e-health literacy with a reliable and valid scale such as eHEALS-E has important implications for practice, ranging from advancing knowledge in the field to informing interventions, optimizing digital health tools, and reducing health inequalities. Our findings show that eHEALS-E is a valid scale that can be used to compare different socio-demographic groups. This means that it makes it possible to uncover factors that influence access to and utilization of digital health resources in different segments of population. This can inform the development and evaluation of targeted interventions aimed at designing tailored health communication strategies and e-health literacy education programs that can improve access and the skills and competencies of vulnerable populations [62]. Understanding the level of e-health literacy in different populations can also enable healthcare providers and organizations to, first, more effectively engage patients in active participation in treatment planning and decision-making and, second, involve patients in the process of co-designing the optimization of digital health tools such as patient portals, telemedicine platforms, etc., which can lead to improved access, usability and actual use of such tools that have the potential to contribute to better health outcomes [63]. To this end, our study to certain extent eliminates the potential concerns of healthcare providers that socio-demographic background of patients might influence how they understand the eHEALS-E scale. Our results clearly show that the quality of the scale is independent of patient’s background, at least in terms of the variables used in this study.

### Limitations and research implications

This study has several limitations at the level of methodological procedures and in terms of the properties of the scale, which should stimulate further research. First, the procedure for obtaining data from SNIPH was rather lengthy, resulting in data that is now almost four years old. Nevertheless, the results should still be relevant, as the trends of searching for and using online health-related information have not, according to the most recent data, [64] changed drastically in the last four years. Moreover, the wording of the items is general enough to accommodate various innovations in the technological landscape. The scale should be validated in other countries, also since Eurostat data shows that Slovenia is in the bottom quarter of EU countries in terms of percentage of individuals using the internet for seeking health-related information [65].

Cooperation with SNIPH allowed for a rigorous revision of the initial eHEALS-E scale, including conducting cognitive interviews, which significantly improved the readability of the items, but it would be important in the future to conduct the interviews on the whole set of items. Moreover, it needs to be noted that cognitive interviews were conducted on the original items in Slovene and the English translation would need to go in a similar procedure for testing the readability of the items.

In terms of scale properties, it could be claimed that it is somewhat long for use in larger research and practical settings. Whether a shorter scale can be developed while maintaining the 6-dimensional structure should be investigated. The correlations between the items suggest that there may be some redundant items, which opens up the possibility for a more parsimonious solution. In particular, with regard to the small distinction between the dimensions Awareness of sources and Recognizing quality and meaning, there is the possibility of either making a better distinction or integrating both into a single dimension. With regard to the Being smart on the Net dimension, we would like to acknowledge that this dynamic component of e-health literacy is highly dependent on advances in digital technologies. As a results, these items may require regular review to ensure their relevance and applicability. As already indicated, the items requiring the self-assessment of knowledge and skills are also likely to be susceptible to social desirability bias [66] and would therefore need further testing.

## Conclusions

E-health literacy is one of the most important skills for individuals in today’s society, where online sources are becoming primary points for addressing health-related questions. In addition to being a valid and reliable scale of e-health literacy, it is crucial that it does not lead to bias when comparing sociodemographic groups. With a minor limitation, the scale ensures an unbiased e-health literacy assessment across different social groups, which is crucial for interventions that aim to reduce health-related inequalities. This ensures that the interventions derived from the assessment of reality are equally valid and effective for everyone, regardless of their sociodemographic background.

## Data Availability

The data that support the findings of this study are available from Slovenian National Institute of Public Health (SNIPH), but restrictions apply to the availability of these data, which were used under license for the current study and so are not publicly available. The data are, however, available from the authors upon reasonable request and with the permission of Slovenian National Institute of Public Health (SNIPH).
